# Antibiotic Resistance Pattern in Intensive Care Units in a Large Referral Hospital in Iran

**DOI:** 10.30699/IJP.2023.1990807.3073

**Published:** 2023-10-15

**Authors:** Samaneh Salarvand, Alireza Abdollahi, Pegah Afarinesh Khaki, Mahsa Norouzi Shadehi, Mohammad Taghi Beigh Mohammadi, Seyed Amir Miratashi Yazdi, Elham Nazar

**Affiliations:** 1 *Department of Pathology, Imam Khomeini Complex Hospital, Tehran University of Medical Sciences, Tehran, Iran*; 2 *Central Laboratory, Imam Khomeini Complex Hospital, Tehran University of Medical Sciences, Tehran, Iran*; 3 *Department of Intensive care medicine, I* *mam Khomeini complex hospital, * *Tehran University of Medical Sciences, Tehran, Iran*; 4 *Department of General Surgery, Sina Hospital, Tehran University of Medical Sciences, Tehran, Iran*; 5 *Department of Pathology, Sina Hospital, Tehran University of Medical Sciences, Tehran, Iran*

**Keywords:** Antibiotic-resistant, Blood culture, Intensive care unit

## Abstract

**Background & Objective:**

Antibiotic resistance, especially in the form of multidrug-resistant (MDR), is a big problem, especially in intensive care units (ICUs). This study aimed to evaluate antibiotic resistance and MDR patterns among patients hospitalized in the ICUs in one of the large referral centers in Iran.

**Methods:**

The present study was conducted at Imam Khomeini Hospital in Tehran (a great referral hospital), which admits critically ill patients requiring ICU services. To determine the rate of positive cultures for resistant strains, the patient’s blood specimens were sent to the laboratory of the hospital for inoculation on proper culture media within 2 hours of extraction. Antimicrobial susceptibility tests were done using the Bauer–Kirby disk diffusion method.

**Results:**

A total of 1,755 samples were collected from the patients to assess microbial strains and antibiotic resistance. The most common microbial strains detected in the cultures extracted from peripheral blood samples were *Klebsiella pneumonia* (22.1%), *Staphylococcus* epidermidis (7.9%) and another coagulase-negative Staphylococcus (15.0%). The antibiogram test showed antibiotic resistance in 1,509 cases, leading to a resistance prevalence rate of 85.9%. The most common antimicrobial resistance observed was against cotrimoxazole (61.7%), ciprofloxacin (51.3%), imipenem (50.0%), and ampicillin (49.6%). The rate of MDR was found to be 96.3%.

**Conclusion:**

In Iran’s ICUs, a significantly high level of antibiotic resistance may be seen especially the MDR pattern, which indicates the need to change the pattern of prescribing and managing these drugs in ICU centers.

## Introduction

Although years have passed since the presentation of clinical guidelines to standardize the prescription of antibiotics, antibiotic resistance is still a major health problem in many societies (1). In 1940, the first generation of antibiotics was introduced to the world, but it did not take long for the gradual emergence of antibiotic resistance to become a significant concern. Over time, the resistance levels increased to the point where, in some cases, the resistance rates approached nearly 100%. This led to the ineffectiveness of antibiotics (2, 3). This issue was especially clear and potentially more important in patients with critical illnesses because these patients require long-term hospital stays and more prescriptions of various antibiotics; in addition, they are more vulnerable to nosocomial infections. Antibiotic resistance has several important and undesirable consequences (4). First of all, this resistance, especially in the case of multidrug-resistant (MDR), faced many problems in the management and control of hospital infections (5), leading to increased healthcare costs and mortality rates in hospitals (6). These problems were significantly felt in patients admitted to intensive care units (ICUs). ICU patients are very vulnerable to antibiotic-resistant infections due to vascular accesses, intubation, and mechanical ventilation, as well as prolonged use of different types of intravenous antibiotics (7). In such patients, the rate of nosocomial infections in ICUs has been estimated to be higher than 50% (8). The prevalence rate of antimicrobial resistance is significantly higher than two- to three-fold in ICU admissions longer than 7 days (9). In this regard, some preventive protocols could control and even reduce the antibacterial resistance rate, such as using a glove, patient isolation, handwashing, and extubating of the patient as soon as possible (10). Despite all this, the rate of resistance to antibiotics is reported to be increasing, especially in developing countries (11), which emphasizes the need to modify antibiotic resistance control guidelines. Also, blood cultures are the most sensitive laboratory test for the detection of bacteremia in patients admitted to ICUs (12). In this regard, the first step is to obtain more accurate and comprehensive information about the rate of antibiotic resistance, as well as identify factors related to it on blood cultures. Accordingly, this study aimed to evaluate the rate of antibiotic resistance and its related factors in the ICU wards of one of the large referral hospitals in Tehran.

## Material and Methods

The present cross-sectional study was conducted at Imam Khomeini Hospital in Tehran, a great referral hospital admitting critically ill patients requiring ICU services. This hospital is a major educational center in the capital of Iran and has 1,500 beds with 8 ICUs (including general ICU, cancer ICU, adult cardiac surgery ICU, emergency ICU, imaging ICU, liver transplantation ICU, neurosurgery ICU, and brain stroke ICU). In the current study, all patients admitted to the ICUs due to different reasons and whose companions or their families signed the consent form were included in the study. The baseline information was collected by reviewing the hospital files using a structured questionnaire. The collected data consisted of demographics, reasons for ICU admission, medical history, and vital signs on admission to the ICUs. To determine the rate of positive cultures for resistant strains, the patient’s blood specimens were extracted from the peripheral veins and central venous lines and sent to the laboratory of the hospital for inoculation on proper culture media within 2 hours of extraction according to the Clinical and Laboratory Standards Institute (CLSI) guidelines (13). Antimicrobial susceptibility tests were done using the Bauer–Kirby disk diffusion method according to CLSI protocols (14).

For statistical analysis, the results were presented as mean ± SD for quantitative variables and were summarized by frequency (percentage) for categorical variables. Continuous variables were compared using the *t* test or Mann-Whitney test whenever the data did not appear to have normal distribution or when the assumption of equal variances was violated across the study groups. The categorical variables were compared using the chi-square test or Fisher’s exact test if required. *P* values less than 0.05 were considered statistically significant. For the statistical analysis, SPSS version 23 (SPSS Inc, Chicago, IL, USA) was used.

## Results

**Fig. 1:**
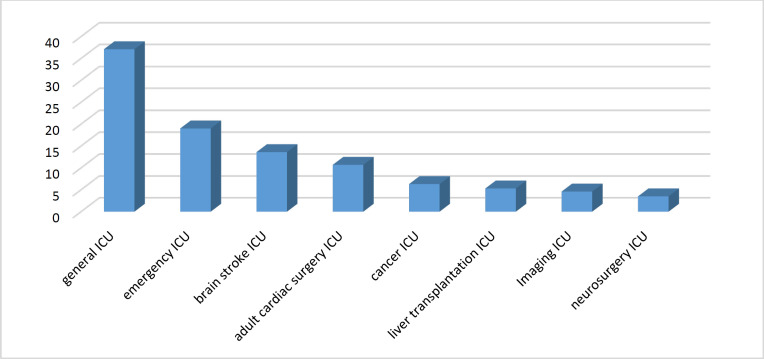
The wards as the sources of the specimens

**Fig. 2:**
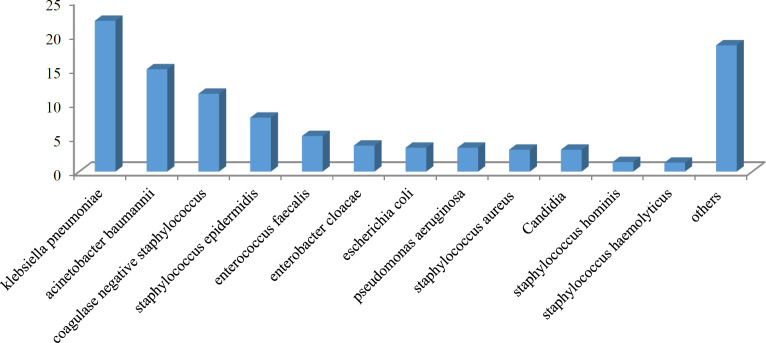
The common microbial strains obtained by blood culture

A total of 1,755 samples were collected from the patients to assess microbial strains and antibiotic resistance. Of those, 914 (52.1%) were male, and 841 (47.9%) were female. As shown in Figure 1, the majority of the samples were obtained from the general ICU (37.1%), followed by the emergency ICU (19.0%) and neurosurgery ICU (13.6%). The most common microbial strains detected in the cultures extracted from peripheral blood samples (Figure 2) were *Klebsiella pneumonia* (22.1%), Staphylococcus epidermidis (7.9%), other coagulase-negative *Staphylococcus* (15.0%), and *Enterococcus faecalis* (3.8%). Of the 1,755 samples extracted, the antibiogram test showed antibiotic resistance in 1,509 cases, leading to a resistance prevalence rate of 85.9%. 

**Fig. 3:**
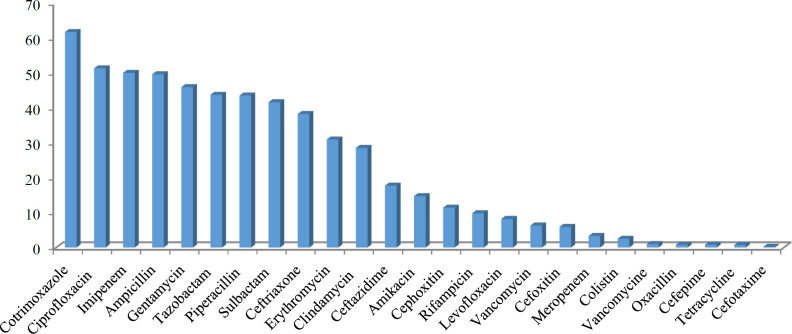
The antibiotic resistance pattern

The most common antimicrobial resistance observed was against cotrimoxazole (61.7%), ciprofloxacin (51.3%), imipenem (50.0%), ampicillin (49.6%), gentamycin (45.9%), tazobactam (43.7%), piperacillin (43.5%), sulbactam (41.6%), ceftriaxone (38.2%), erythromycin (30.9%), clindamycin (28.5%), ceftazidime (17.7%), amikacin (14.7%), and cefoxitin (11.4%) (Figure 3). Regarding resistance to antibiotics among the evaluated cases with drug resistance, the rate of MDR was found to be 96.3%. The highest sensitivity to the antibiotics was specified to the drugs linezolid and vancomycin.

## Discussion

Sepsis is a common reason for mortality in patients admitted to ICUs, and blood cultures could recognize a bacterial pathogen (15). Blood cultures are often performed in ICUs to detect pathogens to guide treatment (16). Thus, blood cultures are essential in critically ill patients to allow them to rapidly administer the proper antimicrobial drugs (17). Blood culture contamination has been related to improper antibiotic use, added laboratory assessments, and increased duration of hospital stay with extra cost for patients (18). In addition, antimicrobial resistance is one of the major public health emergencies worldwide (19). Given the extensive use of injectable antibiotics (especially in ICUs), an increase in the resistance of microbial strains to these drugs can be expected. This problem will be more obvious, especially in the case of countries where drug administration protocols are not based on updated guidelines or where the treatment staff is not obliged to follow these standard protocols. In the last 2 decades, the healthcare system in Iran has experienced tremendous developments regarding the implementation and modification of guidelines based on how to use antibiotics in hospital departments, but in this regard, there are still significant problems in prescribing these antibiotics in our ICUs. According to the present study, we have encountered high microbial resistance regarding some commonly used antibiotics, including sulfonamides, fluoroquinolones, carbapenem antibiotics (especially imipenem), and penicillin family drugs (including ampicillin). The mentioned drugs are widely used as the first line of antibiotic treatment in special care centers in Iran. According to the type of strains detected in this study, it seems that most of these drugs have been used to suppress strains such as *Klebsiella*, *Acinetobacter*, *Staphylococci*, and Enterococci. Of course, the pattern of antibiotic resistance in other societies can be completely different depending on the type of therapeutic and preventive planning against microorganisms. For example, Perez *et al.* found that *A. baumannii* is commonly found in ICUs, whereas in our study, *Klebsiella* was the predominant genus (20). Shrestha *et al.* in Nepal reported that more than 50% of identified *K. pneumoniae* and *Escherichia coli* exhibited cephalosporins and cotrimoxazole resistance (21). In our study, the level of resistance to cotrimoxazole was similar to their findings, but the level of resistance to cephalosporins was lower compared to the mentioned study. In the study by Tran *et al.* in Vietnam (22), antimicrobial resistance in ICUs was commonly found for ceftriaxone (88%), ceftazidime (80%), ciprofloxacin (77%), cefepime (75%), and levofloxacin (72%). Overall, the rate of antimicrobial resistance to any drug was 93%. The 3 commonly isolated microorganisms were *Acinetobacter*, *Klebsiella*, and *Pseudomonas aeruginosa*. In a recent study by Saxena *et al.* (23), *S.* *aureus* and *Klebsiella* species were the most common organisms that were commonly resistant to the beta-lactam group of antibiotics, including cephalosporins and piperacillin-tazobactam. Similar to our study concerning the sensitivity of microbial species to some antibiotics (such as linezolid and vancomycin), in their study, none of the *S. aureus *were resistant to linezolid and vancomycin. In another study in Iran by Bagherian *et al.* in 2022 (24), *E. coli* (68.6%) and *Stenotrophomonas* (41.0%) were the most common bacteria isolated from urinary tract infections and bloodstream infections, respectively and the rate of antibiotic resistance was higher among *Acinetobacter*, *E. coli*, *Stenotrophomonas*, *Enterococcus*, and *Pseudomonas* isolates. Therefore, our main problem regarding resistance to antibiotics in ICU patients is an increase in resistance to antibiotics, including cotrimoxazole, ciprofloxacin, imipenem, ampicillin, and gentamicin, especially in the case of strains of *Klebsiella* and *Acinetobacter* and various strains of *Staphylococcus*. 

Another important finding in this study was a high rate of MDR, which was reported in more than 96% of patients. This finding is commonly reported in other communities as well, which indicates difficulty in controlling hospital infections, especially in ICUs. During the last 3 years and with the spread of the COVID-19 pandemic, the majority of hospital admissions (especially ICU admissions) were dedicated to the patients with COVID-19. In this regard, one of the most important concerns of the treatment staff was a high prevalence of MDR in these patients, and many studies have been published in this regard. For instance, a high prevalence of MDR for *A. baumannii* infections has been well established (25, 26). Of course, regardless of the time frame of the COVID-19 pandemic, a wide range of MDR patterns have been reported from different countries. For example, in the study performed by Khalid Elsorady *et al.* in 2022 (27), MDR organisms were prevalent in 110 (57.0 %) patients. *Klebsiella* species were the most frequent MDROs (26%) with a highest susceptibility to amikacin. Tariq’s study in Afghanistan reported that *Klebsiella*, *E. coli*, *Enterobacter*, and *Staphylococci* were the main pathogens responsible for sepsis in Kabul. Most gram-negative organisms were susceptible to Imipenem and Amikacin and gram-positive organisms were susceptible to Vancomycin (28). Tariq’s study is important because our population is close to Afghans, and our antibiotic susceptibility patterns can be influenced. The selection of the proper antimicrobial drugs is still a major challenge due to the increasing frequency of antimicrobial resistance (29). However, it is important to note that susceptibility rates vary among ICUs and general wards (30). It seems that our medical centers have not been very successful in preventing MDR infections in ICUs; accordingly, it is crucial to reassess and revise our approach in the field of MDR infections. 

## Conclusion

There is a high prevalence of antibiotic resistance, especially MDR, among ICU patients in Iran, where the MDR prevalence is reported to be more than 96%. The most common microbial strains detected in culture samples obtained from the ICU patients were *K. pneumonia* and coagulase-negative *Staphylococcus*. The most common antimicrobial resistance observed was against cotrimoxazole, ciprofloxacin, and imipenem. What causes concern is a high prevalence of MDR, which indicates a need to consider a modification in administration of antibiotics in ICUs.

## Conflict of Interest

There is no conflict to be declared.
